# Too stressed to think? A scoping review of the literature for healthcare educators utilising high acuity clinical scenarios

**DOI:** 10.1186/s12909-024-05949-3

**Published:** 2024-09-11

**Authors:** Jason Betson, Erich C. Fein, David Long, Peter Horrocks

**Affiliations:** 1 Faculty of Health, Australian Catholic University, Building 403, Daniel Mannix Building, 8 – 14 Brunswick St, Fitzroy, VIC 3065 Australia; 2 School of Health and Medical Sciences, Centre for Health Research, University of Southern Queensland, Ipswich, Australia; 3 School of Clinical Sciences, Queensland University of Technology, Brisbane, Australia; 4 School of Psychology and Wellbeing, Centre for Health Research, University of Southern Queensland, Toowoomba, Australia

**Keywords:** Physiological stress, Decision-making, Paramedicine, Education

## Abstract

**Background:**

The practise of paramedicine can be highly stressful particularly where urgent lifesaving decisions need to be made. Traditionally, educators have adopted the approach of placing students in simulated stressful situations as a way of learning to cope with these challenges. It is unclear from the literature whether traditional stress inoculation enhances or hinders learning. This scoping review aims to identify and examine both the peer-reviewed and grey literature reporting physiological stress responses to high-acuity scenarios in paramedicine and cognate healthcare disciplines.

**Methods:**

Adhering strictly to JBI Evidence Synthesis Manual for conducting a scoping review, medical subject headings and areas, keywords and all other possible index terms were searched across EBSCOhost (Medline, CINAHL and APA PsycInfo), Scopus and, PubMed. English language articles both published (peer-reviewed academic papers, reports and conference proceedings) and unpublished (grey literature, Google Scholar reports) were included, and publications citing retrieved articles were also checked.

**Results:**

Searches performed across five electronic databases identified 52 articles where abstracts indicated potential inclusion. From this, 22 articles which reported physiological or psychophysiological responses to stressful scenario-based education were included.

**Conclusion:**

This review identified that an acceptable level of stress during simulation can be beneficial, however a point can be exceeded where stress becomes a hinderance to learning resulting in underperformance. By identifying strategies to moderate the impact of acute stress, educators of paramedic and other healthcare students can utilise high-acuity clinical scenarios to their andragogical armamentarium which has the potential to improve real-world clinical outcomes.

**Supplementary Information:**

The online version contains supplementary material available at 10.1186/s12909-024-05949-3.

## Introduction

In many high-income countries, paramedic education has progressively moved from a post-employment vocational training model of the latter part of the 20th century to now sit firmly within the pre-employment tertiary education sector [1]. This evolution of education enables paramedics to provide high-level emergency care as new-to-practice clinicians in high pressure, time-critical environments [2]. To do this, education providers often utilise high-fidelity simulations to apply clinical or other skills in realistic environments. These simulations, are often comprised of high-acuity scenarios which are designed to depict a high severity of illness or injury [3] requiring rapid medical interventions, which can invoke increased physiological and cognitive stress. It may be the case that if these simulations are too stressful, clinical learnings from them may be lost due to the high stress load the participant is exposed to as shown by Takahashi, et al. [4] who identified higher cortisol levels post stress exposure in university students, which correlated with an increased level of memory impairment and poorer performance.

Links between physiological stress and knowledge application have also been reported in the paramedicine sector. LeBlanc, et al. [5] demonstrated that clinicians made more drug calculation errors following exposure to stressful events, whilst senior paramedics exhibited clinical and documentational vulnerabilities during high-acuity scenarios [6]. In the emerging field of undergraduate paramedicine education research, few studies have explored high-acuity scenario-based education and any associated physiological and cognitive stress. This is in contrast to Harvey, et al. [7], LeBlanc, et al. [6] and, more recently Hase, et al. [8] who have recommended that training in high-acuity areas of medicine should include challenge-promoting interventions specifically relevant to stress mitigation.

In the expanding cohort of university-trained paramedicine students, research on empathy [9], prevention of mental health and psychological disorders [10], workplace violence [11], physical characteristics [12] and pre-employment fitness testing [13] have been published. However, linkages between time critical high-acuity scenario-based education with resultant physiological stress and its potential impacts on cognitive decision-making has not been studied. A recent systematic review explored the physiological responses to acute stress in workers of several occupations, mostly within the human service industry [14]. Whilst this paper draws appropriate conclusions about acute physiological changes leading to performance decrement, possible implications for frontline healthcare workers were limited by a small number of healthcare-based studies included within the review. A gap also exists between self-awareness of one’s own physiological stress and how this may impact clinical judgement. Therefore, the purpose of this review was to better understand the physiological and cognitive stress responses observed in the participants undertaking high-acuity clinical scenarios. By appreciating the existence of contributory factors and how they influence stress, educators of paramedics and other healthcare workers can determine which elements of physiologically and mentally stressful scenario-based education can be considered in the design of their own programs.

## Methods

### Study design

Full systematic literature reviews (SLRs) are generally considered to be the foundation for evidence-based practice, particularly in healthcare [15]. This form of evidence synthesis relies on an extensive base of published literature and is frequently used to validate or refute current practice [16]. However, within the scope of the present study, little extant research reports on physiological changes triggered by high stress learning situations or the consequences this effect has on clinical performance. Given the inter-relationship between acute stress and the degradation of cognitive decision-making ability [7, 17], further research is warranted to characterise this physiological response in undergraduate paramedicine students. In this paper, we employed a scoping review method to explore the extent of published and unpublished literature from cognate heath disciplines to identify key characteristics or factors related to our topic of interest.

Our final protocol was registered on the 21st March 2023, and is publicly available on the Open Science Framework platform (https://osf.io/dxchy/).

### Identifying the research question

This scoping review aims to identify and map the scope of current published literature related to physiological stress responses to high-acuity scenarios and, importantly, identify and analyse the knowledge gaps [18]. To achieve the aim, the following search strategy was employed:

- Participants: higher education students or students in non-university training programs studying towards a recognised healthcare qualification.
- Concept: any study that incorporates clinical scenarios / simulations where physiological (cardiovascular or endocrine) /or psychophysiological data is recorded.
- Context: any undergraduate or postgraduate higher education setting or equivalent non-university training facility for the participants mentioned above.

### Search strategy and eligibility criteria

The latest version [19] of Joanna Briggs Institute (JBI) comprehensive guide for authors conducting a scoping reviews [20] has been followed step-by-step within this review. A search period restriction from 2000 onwards was applied due to the rapid expansion of wearable technology including augmented and virtual reality. To ensure the review examined the acute physiological stress response, it was necessary to focus on articles that assessed markers of stress in real-time as participants were exposed to a stress-inducing task. An initial search was conducted across three prominent databases (Medline, PubMed and Scopus) to determine key terms as a guide to developing a thorough search strategy. From this and with the assistance of a senior research librarian, the secondary search expanded all identified keywords and incorporated medical subject headings (MeSH), major subject areas, and all other possible index terms as noted in the Appendix 1. The protocol incorporated both published (peer-reviewed academic papers, reports and conference proceedings) and unpublished (incorporating theses and dissertations, research and technical reports) evidence but did exclude non-English language articles. Sources were gathered using EBSCOhost (including Medline, CINAHL and APA PsycInfo) Scopus, and PubMed. Google Scholar was also searched as there is a small body of evidence that suggests this search engine produces highly comprehensive results [21, 22] whilst also searching ‘grey literature’ (published informally, non-commercially or remains unpublished), a format neglected by other databases. Selection of papers for inclusion in the study were then undertaken independently by two members of the research team (DL and EF). Finally, any other articles that cited the retrieved articles were also checked using citation alert with the ISI Web of Knowledge (Appendix 1).

### Extracting and charting the data

Data were extracted from the included studies by two reviewers (JB and PH) utilising the JBI template of evidence details, characteristics and results extraction instrument [19]. Initial piloting of the data extraction resulted in some additional data being sought from each publication to allow quality appraisal to occur. This refined data extraction gathered details about study year, study country, study aim, study setting, study design, interventions, and comparators. Additionally, the data included sample size, methods, results, and author recommendations. A third member of the review team (DL) performed an accuracy check.

### Quality assessment

Methodological validity and risk-of-bias appraisal, undertaken concurrently with data charting, was performed via the Mixed Methods Appraisal Tool (MMAT) version 2018 critical appraisal instrument designed by Hong, et al. [23]. For the purpose of this scoping review, an overall score was calculated from mean values of each section to determine methodological quality of each reviewed study (Appendix 4). The authors agreed that no cut-off scores would be applicable as the use of the MMAT was not for inclusion or exclusion purpose, but rather to describe the quality of the of publications reported in this review.

### Synthesis of results

The first author performed narrative synthesis of identified themes and discussed these with the review team for validation. Descriptive results are subsequently reported which align with the intended scope and objectives of this review.

### Ethics statement

Ethical approval was not required for this scoping review.

## Results

**Fig. 1:**
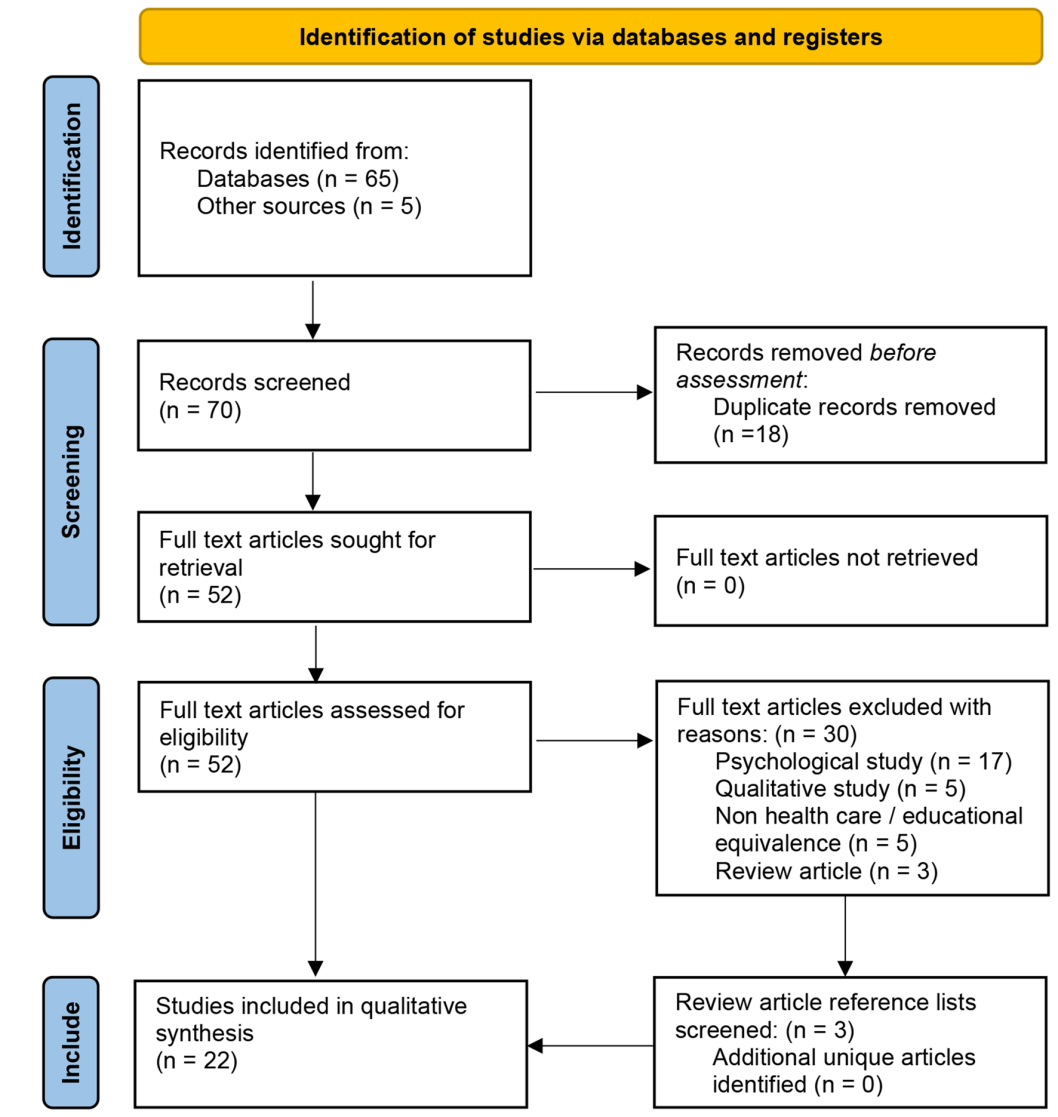
PRISMA flow diagram findings

The search strategy yielded 1427 results, of which 52 remained after title and abstract proofing and duplicate removal (Level 1). Consensus was not reached on seven papers with resolution sought from a third member of the review team (PH) (Appendix 2). Of the 52 studies, 30 were excluded for reasons outlined in Appendix 3. In addition, reference lists of three excluded review articles were checked, although nil additional suitable articles were identified. Unpublished (grey) literature was also assessed with no additional studies deemed suitable for inclusion. Figure 1 illustrates a Preferred Reporting Items for Systematic reviews and Meta-Analyses (PRISMA) flow diagram [24] of the process and Table 1 lists the 22 studies deemed eligible for inclusion.

**Table 1:** Articles eligible for inclusion

Author and Year	Country	Aim, Setting and participants	Methods (design, intervention and comparators)	Results and key findings	Author recommendations
Baker BG, Bhalla A, Doleman B, Yarnold E, Simons S, Lund JN, Williams JP. 2017	United Kingdom	Aimed to compare the stress induced by simulation with clinical environments. Eight anaesthetic trainees in their first 3 years of study.	Objective (heart rate variability (HRV)) and subjective (state trait anxiety inventory (STAI) measures of stress recorded and compared.	Simulation was unable to accurately replicate the stress of the technical procedure performed in theatre. Simulation may induce a level of pre-performance anxiety.	Evaluate participant stress in different simulated environments to assess triggers as a learning tool. Optimize simulation for assessment and stress management training.
Barbadoro P, Brunzini A, Dolcini J, Formenti L, Luciani A, Messi D, Papetti A, Ponzio E, Germani M, Adrario E. 2023.	Italy	Aimed to compare stress responses during high-fidelity (HF) and procedural simulation (PS) for 148 medical students.	Compared self-perceived and biological level (salivary cortisol variations) stress responses for two simulation methods.	Both the psychological and the biological stress levels increased due to simply being in a simulation scenario; opposed to the intrinsic complexity of tasks being performed.	More studies are needed to confirm this trend and to clarify the role of simulated stress response in a long- term learning.
Beltrán-Velasco AI, Bellido-Esteban A, Ruisoto-Palomera P, Clemente-Suárez VJ. 2018.	Spain	Aimed to explore changes in the autonomic stress response of 14 psychology students during a stressful OSCE.	Analysis of HRV in temporal, frequency and non-linear domains, subjective perception of distress strait and academic performance were measured before and after the two different OSCEs.	Produced a large anxiety anticipatory response, a habituation response; autonomic modulation parameters did not correlate with academic performance of these students.	Instruments to measure HRV in real time during OSCE useful increase students’ performance; help students control stress response using biofeedback; help teachers improve simulation experience.
Beltrán-Velasco AI, Ruisoto-Palomera P, Bellido-Esteban A, García-Mateos M, Clemente-Suárez VJ. 2019.	Spain	Aimed to study psychophysiological responses of 15 physiotherapy students during clinical practice and assess their coping style relevant to impact on academic performance.	Analysis of HRV along with six different psychological measurements questionnaires across 11 evaluation points of a three-month clinical placement.	None of the psychophysiological variables analysed were related to academic performance. No habituation / no increased parasympathetic response.	Extending this study to other educational levels could provide valuable information on the stressful events faced and strategies used.
Bialka S, Copik M, Ubych A, Marciniak R, Smereka J, Szarpak L, Misiolek H. 2021.	Poland	Prospective, observational study evaluating the degree of stress in 55 final year medical students during critical care classes.	Before and after scenario, systolic blood pressure (BP), diastolic BP, mean BP, Heart Rate (HR), blood oxygen saturation and saliva stress hormone levels assessed.	BP and HR significantly higher after scenario. Stress hormones remained elevated after scenario for two hours.	High-fidelity simulation induces stress comparable with clinical duties, but it can also lead to underperformance if the stress is too high.
Demaria S, Jr., Bryson EO, Mooney TJ, Silverstein JH, Reich DL, Bodian C, Levine AI. 2010.	United States of America	Aimed to determine if added emotional stress increased anxiety during cardiopulmonary arrest scenario for 25 medical students early in their studies.	Randomised control trial (RCT) with actors scripted to add stress (ES). HR and subjective stress recorded before and after with follow up at six months later.	ES group experienced increased physiological responses and greater anxiety. Six months later, written test scores similar, but ES group participants achieved higher practical competency.	Limited data regarding the effects of stress on educational outcomes in the medical simulation literature. This investigation suggests that there are levels of anxiety that enhance learning.
DeMaria S, Silverman ER, Lapidus KAB, Williams CH, Spivack J, Levine A, Goldberg A. 2016	United States of America	Aimed to describe the physiologic and biochemical stress response induced by simulated patient death for 26 medical students early in their studies.	**H**igh-fidelity simulation (HFS) used with participants randomized to simulated death or survival. HR and salivary stress hormone levels assessed.	Learners experience stress during HFS; however, no detectable difference or negative response to a simulated patient death compared with simulated survival.	Notion that concerns over deliberately permitting the simulated patient to die may be overestimated, authors found no apparent psychological harm
Feeley AA, Feeley IH, McManus R, Lunn JV, Sheehan E, Merghani K. 2022.	Ireland	Aimed to evaluate the cumulative impact of supervision on technical skills and surrogate stress markers in 20 orthopaedic surgical trainees at a Tertiary trauma centre.	Quasi-experimental crossover study evaluating impact of attending supervision on trainee stress response (HRV) during a simulated surgical procedure.	Presence of supervision during simulated surgical performance increased stress response compared to unsupervised task and baseline measurements.	Accretion of technical and non-technical skills may benefit from the use of simulation-based training in surgical residents in both supervised and unsupervised sessions.
Flinn JT, Miller A, Pyatka N, Brewer J, Schneider T, Cao CGL. 2016.	United States of America	To characterize the effects of stress resulting from attending–trainee interaction during surgical skill acquisition for 40 medical students.	RCT – participants observed, encouraged, or criticized by an expert. BP, HR, skin conductance, salivary cortisol, task performance and subjective stress assessed.	Participants who were criticized performed the worst and those who were encouraged performed best. Physiological and subjective measures showed criticized participants experienced the highest level of stress and anxiety.	Teacher–student interaction should not be negatively critical to the point of being appraised as threat-like in nature. Future research investigating the threshold for threat in stress appraisals to allow for more effective teacher-student interactions.
Harvey A, Nathens AB, Bandiera G, LeBlanc VR. 2010.	Canada	Determine extent to which cognitive appraisal affects 13 medical trainee’s physiological and subjective stress responses to high-acuity simulated clinical situations.	RCT - high (HS) and low (LS) stress trauma resuscitation simulations. Subjective and physiological (salivary cortisol) measures compared.	Post-scenario, subjective stress scores, cognitive appraisal and cortisol levels were higher in the HS scenario compared with the LS scenario.	Subjective appraisals of a situation as a threat impairs performance. As such, training for high-acuity events should include interventions targeting stress management skills.
Judd BK, Alison JA, Waters D, Gordon CJ. 2016.	Australia	Aimed to compare acute stress during simulation-based clinical education with that experienced in situ in a hospital- based environment for 33 undergraduate physiotherapy students.	Subjective stress responses, anxiety, continuous HR, and saliva cortisol assessed in simulation using standardized patients and during hospital clinical placements with real patients.	Stress and anxiety were significantly higher in simulation compared with hospital. Physiological stress responses (HR and cortisol) were comparable.	New learners in their clinical education program may benefit from a less stressful simulation environment before a gradual increase in stress demands as they approach clinical practice.
Keitel A, Ringleb M, Schwartges I, Weik U, Picker O, Stockhorst U, Deinzer R. 2011.	Germany	To assess psychological and endocrine stress responses for 34 medical students and relationship between stress reactivity and medical performance in simulation	A counterbalanced cross-over design of three situations. Salivary cortisol, psychological responses medical performance were assessed.	Cortisol increased significantly in both stress conditions and surprisingly correlated significantly with improved medical performance.	Future studies should analyse the educational implications of the massive stress response observed under simulation and if this actually improves clinical performance.
Los, K, Chmielewski J, Cebula G, Bielecki T, Torres K, Luczynski, W. 2021.	Poland	Aimed to determine whether the technical and non-technical skills of medical students undertaking 166 paediatric high-fidelity simulations are related mindfulness and stress.	Standardized simulations were conducted assessing: stress sensation (subjectively and HR / BP) technical and non-technical skills and mindfulness.	Mindfulness of students is associated with non-technical skill i.e., avoiding fixation error. However, did not correlate with alterations in HR and pressure before and after a simulation.	Mindfulness courses included in the medical university curriculum and early career of a young doctor to reduce stress and improve the diagnostic and therapeutic effectiveness of physicians.
MacQuarrie AS, Hunter JR, Sheridan S, Hlushak A, Sutton C, Wickham J. 2022.	Australia	To assess if the clinical performance of 11 paramedicine students and graduates is affected by fatigue, compared with performing the scenario without prior activity.	Randomized crossover design of high-fidelity simulations after either acute physical activity or rest. HR, respiratory rate (RR), BP, and clinical performance assessed.	Participants performed as well, or better, immediately after acute physical activity compared with rest, despite higher physiological exertion.	This pilot study shows physical activity did not result in clinical performance decrements and should be the catalyst for further research in this area.
Martín-Rodríguez F, Castro Villamor MA, López-Izquierdo R, Portillo Rubiales RM, Ortega GJ, Sanz-García A. 2021	Spain	Aimed develop a predictive model (demographic, physiologic, and metabolic) capable of determining which final year medical students (*n* = 163) present high anxiety levels.	Randomized, sham-controlled, blinded trial assessing HR, BP, salivary pH, lactate and subjective stress questionaries before and after simulations.	Predictive demonstrated age and systolic blood pressure found to be protective factors against anxiety. The type of scenario or the role played had no effect on anxiety.	Early identification of which students will have high levels of anxiety could allow the introduction of avoidance measures and improve learning during the medical simulation.
McKay KAC, Buen JE, Bohan KJ, Maye JP. 2010.	United States of America	Prospective descriptive design to compare baseline, acute, and recovery measurements of stress with performance scores of 18 student nurse anaesthetists.	Students simulated induction and intubation sequence. HR, BP sweat levels, salivary α-amylase and subjective stress levels assessed before and after.	Low performers increased stress and performed poorly, high performers increased stress and performed superbly, and moderate performers had modest stress and performed moderately.	Obtaining more knowledge related to the impact of acute stress on performance can only benefit and improve educational programs and lead to the development of strategies to help students succeed.
Nakayama N, Arakawa N, Ejiri H, Matsuda R, Makino T. 2018.	Japan	This study sought to clarify stress changes throughout the progression of different phases of a simulation for 74 nursing students.	HRV and subjective responses recorded across 4 phases of simulation - the break, patient care, reporting, and debriefing.	The reporting phase involved high objective and subjective stress. The debriefing phase did not significantly differ from the break phase for objective or subjective stress.	It may be possible that the educator’s evaluative attitude increased students’ stress. Therefore, a stress intervention during the reporting phase might further improve student performance during that phase.
Palekar TJ, Mokashi MG, Anwer S, Kakrani AL, Khandare SD, Alghadir AH. 2015	Saudi Arabia	Aimed to assess if galvanic skin resistance-aided biofeedback (GSRBF) could reduce stress in 43 physiotherapy students.	A pretest-posttest quasi-experimental study assessing stress metrics (HR, RR, BP, and perceived stress) following GSRBF training.	GSRBF training was found to produce a significant reduction in the physiological response and perceived stress in physiotherapy students.	Further, controlled research should be conducted to verify these findings and to identify if long term GSRBF is an effective stress reducing training protocol.
Park HJ, Choi D, Park HA, Lee CA. 2022.	Republic of Korea	Aims to evaluate 132 nursing students’ stress levels during CPR simulation training and to determine the correlation between individual personality traits and stress levels.	Prospective observational study measuring HRV via a smartwatch and any correlation between personality and stress.	A weak negative correlation was observed between the agreeableness personality trait and stress measurements.	More effective training can be developed when individual perception, opinions, and experiences are considered within individuals’ acceptable stress levels.
Rieber N, Betz L, Enck P, Muth E, Nikendei C, Schrauth M, Werner A, Kowalski A, Zipfel S. 2009.	Germany	The purpose of this study was to quantitatively evaluate stress and motivation in both students and standardised patients (SPs) during scenarios (*n* = 44 medical students)	Stress and motivation in both students and SPs during scenarios. HRV measured continuously; motivation questionnaire before scenario.	HRV lower in both students and SPs during the scenarios. Motivation increased for scenario involving psychosomatic illness but decreased for somatic.	Future studies to consider subjective tension and motivation prior to and after scenario to elucidate individual contributions made by knowledge, motivation and stress.
Stecz P, Makara-Studzińska M, Białka S, Misiołek H. 2021.	Poland	Study aimed to assess stress parameters of 56 undergraduate anaesthesiology students	Prospective observational study assessing psychological (anxiety and stress state), physiological (HR, BP), immunological, and humoral levels of stress during high-fidelity simulation training.	No clear relationships were found between biological stress and trait anxiety. Stress markers varied depending on the assigned roles; however, the trajectories of responses were similar.	Medical students were generally resistant to acute stress. Best practice should involve management of students’ wellbeing, i.e., emotional distress reduction after simulation or switching the assigned roles.
Tramèr L, Becker C, Hochstrasser S, Marsch S, Hunziker S. 2018.	Switzerland	Study aimed to analyse electrocardiogram (ECG) alterations in 126 medical student rescuers and its association with gender and CPR performance.	Prospective, observational simulator study using ECG output before, during and after CPR. HR, HRV, and ST- and T-wave morphology analysed.	HR increased significantly during resuscitation, from values before resuscitation and decreased after resuscitation. T-wave and ST-segment abnormalities observed in approx. 30% of participants.	Cardiopulmonary resuscitation caused significant ECG alterations in healthy medical students with ST-segment and T-wave abnormalities, with more pronounced effects in females.

From the twenty-two studies identified that met the inclusion criteria, the majority originated from Europe and North America. Only one study [25] involved paramedics or paramedicine students. Twelve studies involved medical or surgical trainees [7, 26–36], five studied nursing or nurse anaesthetist students [37–41], three studies involved physiotherapy students [42–44], and one study examined psychology students [45].

No studies were identified that warranted exclusion based on major methodological flaws on any significant risk of bias. However, study design flaws and lower levels of evidence were common. Of most concern were poorly described methodologies and under-powered sample sizes incapable of producing statistically significant results (see Appendix 4 for tabulated quality assessment results). Examining the methodology used, sixteen studies adopted a mixed methods approach and six utilised a quantitative method. Randomised controlled trials were reported in seven papers and a battery of different metrics were recorded across the studies. Heart rate variability and salivary cortisol levels were the most frequently reported objective data, whilst the state trait anxiety inventory was the most common subjective measure (see Table 2).

**Table 2:** Study metrics of stress

	Studies utilising this metric
Physiological	
• Heart rate or heart rate variability	[25, 27–31, 33–37, 39–45]
• Blood pressure (systolic / diastolic / mean arterial)	[25, 27, 31, 33, 34, 41, 44]
• Skin conductance / skin response	[31, 44]
• Respiration rate	[25, 44]
• *Other*: temperature [34], oxygen saturation [27], electrocardiogram alterations [36], calories burned [30], capillary lactate [34].	
• Saliva:	
cortisol	[7,26 , 27,29 , 32, 41, 43]
secretory immunoglobulin A	[27, 41]
testosterone	[27, 41]
α-amylase	[3838,^41^]
Other: dehydroepiandrosterone [29], pH [34], protein levels [41].	
**Subjective**	
• State trait anxiety inventory	[7, 26, 28, 31, 34, 37, 38, 41, 43]
• Visual analogue scales of stress and anxiety	[32, 43]
• Perceived stress scale	[42, 44]
• Other: NASA-Task Load Index [26], Subjective scale of distress [42], Life engagement test [42], The coping flexibility scale [42], Framingham type a behaviour scale [42], NEO five-factor inventory [,^42^42], Dundee stress state questionnaire [44], Cognitive appraisal [7], Motivation [41].	
**Task performance / clinical outcome**	[31, 38]

In general terms, the twenty-two included studies had similar aims centring around determining how successful high stress simulation could be at replicating clinical experience. Most studies involved both male and female participants with samples sizes ranging from *n* = 8 to *n* = 166, with a mean of *n* = 53 and a median of *n* = 33. Multiple studies assessed and compared stress levels of participants in different situations, and then used these results to determine if stress had affected clinical performance. Other studies used similar data to improve education or training with the aim of ultimately increasing student confidence and performance. The key outcomes from the included studies are summarised in Table 3.

**Table 3:** Key study findings

Key finding	Studies reporting
• Simulation able to replicate real world clinical situation / clinical stress:	
Yes No	[27, 29] [32, 37, 43]
• Pre-performance anxiety / anticipatory anxiety reported	[26, 34, 37, 41, 45]
• Increased psychological stress during and / or post scenario	[7, 26, 28, 31–34, 38, 39, 43]
• Increased biological stress during and / or post scenario	[7, 25–27, 29–33, 35, 36, 38, 39, 43]
• Habituation observed:	
Yes No	[31, 45] [42, 45]
• Stress response related to academic performance:	
Yes No	[7, 30–32, 38] [25, 33, 42]

## Discussion

This review identified studies exploring physiological responses of participants undertaking high stress scenario-based education or training. While acknowledging much of the evidence was of low methodological quality [46] and therefore limits generalisability, the results still provide some useful insights that may be used to inform educators of future paramedics and other healthcare workers.

One of the key findings from this review was the identification of pre-performance or anticipatory anxiety exhibited across multiple studies [37, 38, 45]. This is an area where simulation may not replicate clinical work. Students aware of an upcoming scenario well in advance have ample time to prepare and mount a physiological stress response. This could be controlled if students were given little notice, however this was not commonly reported in the studies. Healthcare educators utilising scenario-based education may choose to restrict prior notification as a means of assessing any changes in the stress response amongst their students. In high-acuity clinical work, paramedics usually have little time to prepare, which may reduce the anticipatory stress response. Potentially this may be seen as positive, as stress has been demonstrated to lead to poorer performance is some paramedic research [5, 6]. However, the evident stress of attending high-acuity cases must also be considered and its impact on performance. In the context of anticipatory anxiety predicting future performance, little research has examined its immediate effect on motor task performance.

The reviewed publications also provide contradictory support for simulation as a tool to replicate the psychophysiological stress of high-acuity clinical work. Baker, et al. [37], in a study with trainees in the highly specialised field of anaesthetics, found simulation was able to replicate the physical and procedural forms of clinical work, however it was unable to replicate the intrinsic level of stress the trainees exhibited when working with a real patient in an operating theatre. These results are potentially influenced by small participant numbers (*n* = 8) and may also be applicable to highly specialised and highly technical fields such as anaesthesia. For paramedicine, contemporary literature [47] highlights simulation allowing for the training of skills that are rarely needed or rarely practiced in the field and supports recommendation made by O’Meara, et al. [2]. For educators of paramedics and other healthcare workers, simulating high-acuity situations is a crucial way to expose students to potential clinical scenarios they may face early in their career. In designing programs of study, careful use of stress-inducing high-acuity simulation can be a beneficial but can also lead to continued underperformance if the stress is chronically too high.

Barbadoro, et al. [26] and Judd, et al. [43] found simulation provided a higher level of stress in their participants when compared to equitable clinical work, whilst Demaria, et al. [28] found that high stress situations can be beneficial for learning. This benefit of high stress learning was also supported by the work of Keitel, et al. [32], who found increased levels of the key stress hormone cortisol correlated with improved memory retention and medical performance amongst medical trainees. An increased stress response was also reported when supervisors or assessors were present within the simulation [30, 31] and, unsurprisingly, vital signs as a measure of physiological stress, increased when the simulation itself was exertive [25, 27, 36]. The stress placed on students involved in high-acuity simulation must be further studied to allow educators to determine what level of anxiety may enhance learning without impeding performance.

Performance ability or academic standing was also found to correlate with stress. McKay, et al. [38] found low performers increased stress and performed poorly, whereas high performers also increased stress but performed superbly in a cohort of student nurses. Paramedicine courses may show similar trends, with students likely to self-assess their academic abilities and stress tolerance. Educators could potentially use real-time learning analytics to offer tailored support and guidance based on live biometric data, proactively aiding students. This would be resource intensive for academics with large student numbers; but in smaller cohorts, the individual feedback around acceptable stress to achieve simulated clinical success may enhance the education program.

Lacking from the literature is a detailed discussion of a variety of variables related to student stress responses from the level of acuity of a scenario. These confounding variables, such as pre-established coping styles and perceived stress intensity within participants, need to be quantified to accurately gauge the success of any interventions aimed at alleviating the stress response, and in examining what levels of anxiety may enhance learning without impeding performance.

### Limitations

Whilst the systematic approach to this scoping review explored multiple electronic bibliographic repositories, there is potential some contemporary conference proceedings, dissertations and theses, along with grey literature not readily available in electronic databases or Google Scholar, may have been missed. Non-English literature may have added value to this review and we attempted to seek translated papers where possible, but we accept that some results may have been missed through this process.

### Directions and recommendations for future research

This scoping review identified inconsistencies and varying methodologies for the assessment of participant stress response in scenario-based education. Recommendations should be developed to identify gold standard quantification of psychophysiological stress responses during high stress scenarios. This would then allow meta-analysis or other systematic synthesis of data to be undertaken to accurately determine any inter-relationship between acute stress and the degradation of cognitive decision-making for healthcare education programs. In addition, variables related to student stress responses from the level of acuity of a scenario should be investigated. For example, individual differences in participants such as pre-established coping styles and strategies, perceived stress intensity, perceived control of stress or coping skill, as well as context specific stressors such as the outcomes associated with scenario performance (e.g., high stakes versus low stakes outcomes) may all be important variables for future research.

## Conclusion

The studies identified in this scoping review have shown high-acuity simulation can induce stress comparable with paramedicine clinical practice. For educators, understanding the factors or elements which contribute to an acceptable level of stress can allow participants the opportunity to fail and learn from their errors during simulation. This further provides opportunities to improve student outcomes in paramedicine and other healthcare education by facilitating high-acuity clinical scenarios that challenge students without inducing stress levels that hinder performance. As educational and wearable technology further evolves, utilisation of real-time biofeedback through passive measurement devices also hold promise as an intervention to reduce the negative effects of acute physiological stress during training scenarios.

## Electronic supplementary material

Below is the link to the electronic supplementary material.

Supplementary Material 1

## Data Availability

Data supporting Fig. 1; Tables 1, 2 and 3 and available within the Supplementary Information (Appendices).
