# Supplementary material for: Too stressed to think? A scoping review of the literature for healthcare educators utilising high acuity clinical scenarios
Source: BMC Med Educ. 2024 Sep 11;24:990. doi: 10.1186/s12909-024-05949-3 (PMC11391698; doi:10.1186/s12909-024-05949-3)
Supplement: Supplementary file 1 — Supplementary Material 1 [file 12909_2024_5949_MOESM1_ESM.docx]

**Appendix 1: Scoping Review Search strategy**

**Research question:** Physiological responses to high-acuity clinical scenarios – what can educators of undergraduate Paramedicine students learn from the literature?

|  | **Concept 1** | **AND** | **Concept 2** | **AND** | **Concept 3** | **AND** | **Concept 4** |
| --- | --- | --- | --- | --- | --- | --- | --- |
| **Initial search using key words:** | **OR** “simulat*”  **OR** “practical”  **OR** “scenario”  **OR** “clinical” |  | **OR** “student”  **OR** “undergraduate”  **OR** “educat*”  **OR** “train*” |  | **OR** “health”  **OR** “medic*”  **OR** “nurs*” |  | **OR** “physiological”  **OR** “psychophysiological”  **OR** “stress” |
| **Database:** | **MEDLINE** |  |  | | | | |
| **Secondary search using key words and additional MeSH terms listed** | (High Fidelity Simulation Training) OR (Simulation) OR (Clinical Competence) OR (Simulation Training/*organization & administration) OR (Interprofessional Relations) | **AND** | (Education) OR (Educational Measurement) OR (Students) OR (Adult) OR (Undergraduate) OR (Young adult) OR (Specialty /Education) OR (Interdisciplinary Placement) | **AND** | (Medical) OR (Students, Medical/*psychology) OR (Students, Nursing/*psychology) OR (Professional) OR (Physicians) | **AND** | (Adaption) OR (Psychological) OR (Epidemiology) OR (Health Surveys) OR (Burnout) |
| **Database:** | **CINAHL** |  |  |  |  |  |  |
| **Secondary search using key words and additional major subjects listed** | (Competency-based) OR (Clinical Competence) OR (Student Experiences) OR (Patient Simulation) OR (Student Performance Appraisal) | **AND** | (Education, Clinical) OR (Education, Nursing) OR (Education) OR (Learning) OR (Learning Methods) | **AND** | (Students, Nursing) OR (Students, Medical) OR (Nonprofessional) OR (Emergency Medical Technicians) OR (Military Personnel) | **AND** | (Stress, Psychological) OR (Empathy) OR (Student Attitudes) OR (Anxiety – Evaluation) OR (Stress, Occupational) OR (Medical Psychosocial Factors) |
| **Database:** | **APA PsycInfo** |  |  | | | | |
| **Secondary search using key words and additional major subjects listed** | (Clinical Methods Training) OR High Fidelity Simulation Training) OR (Simulation) OR (Clinical Competence) | **AND** | (Medical Education) OR (Nursing Education) OR (Education) OR (Learning) OR (Learning Methods) | **AND** | (Medical Students) OR (College Students) OR (Nurses) OR (Military Veterans) OR (Physicians) OR (Medical Residency) | **AND** | (Student Attitudes) OR Adaption) OR (Psychological) OR (Occupational Stress) OR (Academic Stress) OR (Stress Reactions) |
| **Database:** | **SCOPUS** |  |  | | | | |
| **Key words searched and Limiters applied to TITLE-ABS-KEY search** | AND  ( LIMIT-TO ( SRCTYPE ,  "j" ) )  AND  ( LIMIT-TO ( DOCTYPE ,  "ar" ) )  AND  ( LIMIT-TO ( LANGUAGE ,  "English" ) )  AND  ( LIMIT-TO ( EXACTKEYWORD ,  "Human" )  OR  LIMIT-TO ( EXACTKEYWORD ,  "Article" ) )  AND  ( EXCLUDE ( SUBJAREA ,  "ENVI" )  OR  EXCLUDE ( SUBJAREA ,  "BIOC" )  OR  EXCLUDE ( SUBJAREA ,  "NEUR" )  OR  EXCLUDE ( SUBJAREA ,  "PHAR" )  OR  EXCLUDE ( SUBJAREA ,  "AGRI" ) )  AND  (  LIMIT-TO ( OA ,  "all" ) )  AND  ( EXCLUDE ( SUBJAREA ,  "COMP" )  OR  EXCLUDE ( SUBJAREA ,  "ARTS" )  OR  EXCLUDE ( SUBJAREA ,  "ENGI" ) ) | | | | | | |
| **Database:** | **PubMed** |  |  | | | | |
| **Key words searched and Limiters applied to TITLE and ABSTRACT search** | Filters applied: Full text, Books and Documents, Clinical Trial, Meta-Analysis, Randomized Controlled Trial, Review, Systematic Review, Preprints, English. | | | | | | |

**MEDLINE complete** search conducted 20^th^ March 2023 – returned 250 search results. 20 articles identified for further review based on title and abstract.

**CINAHL** search conducted 20^th^ March 2023 – returned 330 search results. 14 additional / unique articles identified for further review based on title and abstract.

**APA PsycInfo** search conducted 20^th^ March 2023 – returned 341 search results. 3 additional / unique articles identified for further review based on title and abstract.

**SCOPUS** search conducted 20^th^ March 2023 – returned 366 search results. 5 additional / unique articles identified for further review based on title and abstract.

**PubMed** search conducted 20^th^ March 2023 – returned 140 search results. 4 additional / unique articles identified for further review based on title and abstract.

**Google Scholar** search conducted 20^th^ March 2023 – returned 72,800 search results. A search through the top 200 results identified a further 6 additional / unique articles for further review based on title and brief abstract. This search was re-run several times across 3 different internet browsers (Google Chrome, Safari and Mozilla Firefox) using slightly different key word combinations, each returning a similar number of search results. Each time, the top 200 results were scanned, but no further unique articles were identified.

Inclusion criteria:

- Participants: higher education students or students in non-university training programs studying towards a recognised healthcare qualification.
- Concept: any study that incorporates clinical scenarios / simulations where physiological (cardiovascular or endocrine) /or psychophysiological data is recorded.
- Context: any undergraduate or postgraduate higher education setting or equivalent non-university training facility for the participants mentioned above.

Exclusion criteria (note how many were excluded in each category):

- Studies that gathered pure psychological data, not psychophysiological data
- Pure qualitative studies with no quantitative (objectively recorded) data
- Non healthcare education related
- Other literature reviews

**Electronic databases and search engines:** Medline, CINAHL, APA PsycInfo, Scopus, PubMed and Google Scholar.

**MEDLINE Search strategy**:

(“simulat*”) OR (“practical”) OR (“scenario”) OR (“clinical”) AND (“student”) OR (“undergraduate”) OR (“train*) OR (“educat*”) AND (“medic*”) OR (“health”) OR (“nurs*”) AND (“physiological”) OR (“psychophysiological”) OR (“stress”)

**Limiters**

- English Language
- Full text

**CINAHL Search strategy:**

(“simulat*”) OR (“practical”) OR (“scenario”) OR (“clinical”) AND (“student”) OR (“undergraduate”) OR (“train*) OR (“educat*”) AND (“medic*”) OR (“health”) OR (“nurs*”) AND (“physiological”) OR (“psychophysiological”) OR (“stress”)

**Limiters**

- English Language
- Full text
- Human

**APA PyscInfo Search strategy**:

(“simulat*”) OR (“practical”) OR (“scenario”) OR (“clinical”) AND (“student”) OR (“undergraduate”) OR (“train*) OR (“educat*”) AND (“medic*”) OR (“health”) OR (“nurs*”) AND (“physiological”) OR (“psychophysiological”) OR (“stress”)

**Limiters**

- English Language
- Full text

**Scopus Search strategy**:

**Search and limiters:**

( TITLE-ABS-KEY ( "simulat"  OR  "practical"  OR  "scenario"  OR  "clinical" )  AND  TITLE-ABS-KEY ( "student"  OR  "undergraduate"  OR  "train" )  AND  TITLE-ABS-KEY ( "medic"  OR  "health"  OR  "nurs" )  AND  TITLE-ABS-KEY ( "physiological"  OR  "psychophysiological" ) )  AND  ( LIMIT-TO ( SRCTYPE ,  "j" ) )  AND  ( LIMIT-TO ( DOCTYPE ,  "ar" ) )  AND  ( LIMIT-TO ( LANGUAGE ,  "English" ) )  AND  ( LIMIT-TO ( EXACTKEYWORD ,  "Human" )  OR  LIMIT-TO ( EXACTKEYWORD ,  "Article" ) )  AND  ( EXCLUDE ( SUBJAREA ,  "ENVI" )  OR  EXCLUDE ( SUBJAREA ,  "BIOC" )  OR  EXCLUDE ( SUBJAREA ,  "NEUR" )  OR  EXCLUDE ( SUBJAREA ,  "PHAR" )  OR  EXCLUDE ( SUBJAREA ,  "AGRI" ) )  AND  (  LIMIT-TO ( OA ,  "all" ) )  AND  ( EXCLUDE ( SUBJAREA ,  "COMP" )  OR  EXCLUDE ( SUBJAREA ,  "ARTS" )  OR  EXCLUDE ( SUBJAREA ,  "ENGI" ) )

**PubMed Search strategy**:

( "simulat" OR "practical" OR "scenario" OR "clinical" ) AND ( "student" OR "undergraduate" OR "train" ) AND ( "medic" OR "health" OR "nurs" ) AND ( "physiological" OR "psychophysiological" OR “Stress”)

**Limiters**

- English Language
- Full text
- Exclude Medline

**Google Scholar Search strategy**:

(simulation OR scenario) (education OR undergraduate OR training)

(health OR medical) (physiological OR psychophysiological OR stress)

**Appendix 2: Articles reviewed for inclusion**

**52 Articles for further review (ER):**

|  | Article | Include | Exclude | Exclude reason | Unsure |
| --- | --- | --- | --- | --- | --- |
| 1 | Acharya J, Sahani S. 2022. Coping up with Stress as a Medical Student. JNMA; journal of the Nepal Medical Association. 60(248):416-418. |  | X | pure psychological data, not psychophysiological data |  |
| 2 | Al-Ghareeb AZ, Cooper SJ, McKenna LG. 2017. Anxiety and Clinical Performance in Simulated Setting in Undergraduate Health Professionals Education: An Integrative Review. Clinical Simulation In Nursing. 13(10):478-491. |  | X | pure psychological data, not psychophysiological data |  |
| 3 | Baker BG, Bhalla A, Doleman B, Yarnold E, Simons S, Lund JN, Williams JP. 2017. Simulation fails to replicate stress in trainees performing a technical procedure in the clinical environment. Medical Teacher. 39(1):53-57. | X |  |  |  |
| 4 | Barbadoro P, Brunzini A, Dolcini J, Formenti L, Luciani A, Messi D, Papetti A, Ponzio E, Germani M, Adrario E. 2023. Stress responses in high-fidelity simulation and standard simulation training among medical students. BMC medical education. 23(1):116. | X |  |  |  |
| 5 | Beltrán-Velasco AI, Bellido-Esteban A, Ruisoto-Palomera P, Clemente-Suárez VJ. 2018. Use of Portable Digital Devices to Analyze Autonomic Stress Response in Psychology Objective Structured Clinical Examination. Journal of Medical Systems. 42(2):35. |  | X | Non healthcare / medical education related |  |
| 6 | Beltrán-Velasco AI, Ruisoto-Palomera P, Bellido-Esteban A, García-Mateos M, Clemente-Suárez VJ. 2019. Analysis of Psychophysiological Stress Response in Higher Education Students Undergoing Clinical Practice Evaluation. Journal of medical systems. 43(3):68. | X |  |  |  |
| 7 | Bialka S, Copik M, Ubych A, Marciniak R, Smereka J, Szarpak L, Misiolek H. 2021. Effect of high-fidelity simulation on alpha-amylase activity and concentrations of secretory immunoglobulin class A, cortisol, and testosterone among medical students. Endocrine. 73(2):431-438. | X |  |  |  |
| 8 | Brasil GdC, Lima LTB, Cunha EC, Cruz FOdAMd, Ribeiro LM. 2021. Stress level experienced by participants in realistic simulation: a systematic review. Revista Brasileira de Enfermagem. 74. |  | X | pure psychological data, not psychophysiological data |  |
| 9 | Buller MJ, Welles AP, Friedl KE. 2018. Wearable physiological monitoring for human thermal-work strain optimization. J Appl Physiol (1985). 124(2):432-441. eng. |  | X | Not related to higher education or equivalent students |  |
| 10 | Demaria S, Jr., Bryson EO, Mooney TJ, Silverstein JH, Reich DL, Bodian C, Levine AI. 2010. Adding emotional stressors to training in simulated cardiopulmonary arrest enhances participant performance. Medical education. 44(10):1006-1015. | X |  |  |  |
| 11 | DeMaria S, Silverman ER, Lapidus KAB, Williams CH, Spivack J, Levine A, Goldberg A. 2016. The impact of simulated patient death on medical students' stress response and learning of ACLS. Medical teacher. 38(7):730-737. | X |  |  |  |
| 12 | Feeley AA, Feeley IH, McManus R, Lunn JV, Sheehan E, Merghani K. 2022. Evaluating the Impact of Supervision on Surgical Trainees Stress Response During Simulated Surgical Procedures; A Crossover Randomized Trial. Journal of Surgical Education. 79(6):1379-1386. |  | X | pure psychological data, not psychophysiological data |  |
| 13 | Flinn JT, Miller A, Pyatka N, Brewer J, Schneider T, Cao CGL. 2016. The effect of stress on learning in surgical skill acquisition. Medical teacher. 38(9):897-903. | X |  |  |  |
| 14 | Georgiou K, Larentzakis A, Papavassiliou AG. 2017. Surgeons' and surgical trainees' acute stress in real operations or simulation: A systematic review. The Surgeon. 15(6):355-365. |  | X | pure psychological data, not psychophysiological data |  |
| 15 | Gürdil Yilmaz S, Yıldız Karadeniz E, dem Lafçi D. 2022. Clinical‐practice stress levels and factors affecting these on first‐year nursing students. Perspectives in Psychiatric Care. 58(4):3009-3015. |  | X | pure psychological data, not psychophysiological data |  |
| 16 | Harvey A, Nathens AB, Bandiera G, LeBlanc VR. 2010. Threat and challenge: cognitive appraisal and stress responses in simulated trauma resuscitations. Medical Education. 44(6):587-594. | X |  |  |  |
| 17 | Hogg G, Miller D. 2016. The effects of an enhanced simulation programme on medical students' confidence responding to clinical deterioration. BMC medical education. 16:161. |  | X | pure psychological data, not psychophysiological data |  |
| 18 | Judd BK, Alison JA, Waters D, Gordon CJ. 2016. Comparison of Psychophysiological Stress in Physiotherapy Students Undertaking Simulation and Hospital-Based Clinical Education. Simulation in Healthcare. 11(4):271-277. |  | X | pure psychological data, not psychophysiological data |  |
| 19 | Keitel A, Ringleb M, Schwartges I, Weik U, Picker O, Stockhorst U, Deinzer R. 2011. Endocrine and psychological stress responses in a simulated emergency situation. Psychoneuroendocrinology. 36(1):98-108. | X |  |  |  |
| 20 | Kharasch M, Aitchison P, Pettineo C, Pettineo L, Wang EE. 2011. Physiological Stress Responses of Emergency Medicine Residents During an Immersive Medical Simulation Scenario. Disease-a-Month. 57(11):700-705. |  | X | pure psychological data, not psychophysiological data |  |
| 21 | Łoś K, Chmielewski J, Cebula G, Bielecki T, Torres K, Łuczyński W. 2021. Relationship between mindfulness, stress, and performance in medical students in pediatric emergency simulations. GMS journal for medical education. 38(4):Doc78. | X |  |  |  |
| 22 | Macdougall L, Martin R, McCallum I, Grogan E. 2013. Simulation and stress: acceptable to students and not confidence-busting. The clinical teacher. 10(1):38-41. |  | X | pure psychological data, not psychophysiological data |  |
| 23 | MacQuarrie AS, Hunter JR, Sheridan S, Hlushak A, Sutton C, Wickham J. 2022. Paramedic Student Clinical Performance During High-Fidelity Simulation After a Physically Demanding Occupational Task: A Pilot Randomized Crossover Trial. Simulation in Healthcare. 17(4):234-241. | X |  |  |  |
| 24 | Martín-Rodríguez F, Castro Villamor MA, López-Izquierdo R, Portillo Rubiales RM, Ortega GJ, Sanz-García A. 2021. Can anxiety in undergraduate students in a high-fidelity clinical simulation be predicted? A randomized, sham-controlled, blinded trial. Nurse Educ Today. 98:104774. eng. | X |  |  |  |
| 25 | McKay KAC, Buen JE, Bohan KJ, Maye JP. 2010. Determining the Relationship of Acute Stress, Anxiety, and Salivary α-Amylase Level With Performance of Student Nurse Anesthetists During Human-Based Anesthesia Simulator Training. AANA Journal. 78(4):301-309. | X |  |  |  |
| 26 | McKerrow I, Carney PA, Caretta-Weyer H, Furnari M, Miller Juve A. 2020. Trends in medical students' stress, physical, and emotional health throughout training. Medical education online. 25(1):1709278. |  | X | pure psychological data, not psychophysiological data |  |
| 27 | Mendes SS, Salvi CPP, Moraes BFM, De Martino MMF. 2019. INSTRUMENTS FOR THE EVALUATION OF STRESS IN NURSING STUDENTS. Journal of Nursing UFPE / Revista de Enfermagem UFPE. 13(3):829-838. |  | X | pure psychological data, not psychophysiological data |  |
| 28 | Morrell N, Ridgway V. 2014. Are we preparing student nurses for final practice placement? British Journal of Nursing. 23(10):518-523. |  | X | pure psychological data, not psychophysiological data |  |
| 29 | Mueller G, Moloff A, Wedmore I, Schoeff J, LaPorta AJ. 2012. High intensity scenario training of military medical students to increase learning capacity and management of stress response. Journal of special operations medicine : a peer reviewed journal for SOF medical professionals. 12(2):71-76. |  | X | pure psychological data, not psychophysiological data |  |
| 30 | Nachiappan M, Bikramjit P, Aung WT, Htoo HKS, Sudipta P. 2020. The impact of stressors on the learning outcome of high-fidelity patient simulation in undergraduate medical students. The Medical journal of Malaysia. 75(3):209-215. |  | X | pure psychological data, not psychophysiological data |  |
| 31 | Nakayama N, Arakawa N, Ejiri H, Matsuda R, Makino T. 2018. Heart rate variability can clarify students' level of stress during nursing simulation. PLoS ONE. 13(4). | X |  |  |  |
| 32 | Norouzi N, Imani B. 2021. Clinical Education Stressors in Operating Room Students: A Qualitative Study. Investigacion & Educacion en Enfermeria. 39(1):97-108. |  | X | pure psychological data, not psychophysiological data |  |
| 33 | Palekar TJ, Mokashi MG, Anwer S, Kakrani AL, Khandare SD, Alghadir AH. 2015. Effect of Galvanic Skin Resistance-Aided Biofeedback Training in Reducing the Pulse Rate, Respiratory Rate, and Blood Pressure Due to Perceived Stress in Physiotherapy Students. / Fizyoterapi Öğrencilerinde Algılanan Strese Bağlı Nabız Hızı, Solunum Hızı ve Kan Basıncının Azaltılmasında Galvanik Deri Direnci Destekli Bio-Geri Bildirim Eğitiminin Etkisi. Turkish Journal of Physical Medicine & Rehabilitation / Turkiye Fiziksel Tip ve Rehabilitasyon Dergisi. 61(2):116-119. | X |  |  |  |
| 34 | Park HJ, Choi D, Park HA, Lee CA. 2022. Nurse evaluation of stress levels during CPR training with heart rate variability using smartwatches according to their personality: A prospective, observational study. PLoS ONE. 17(6). | X |  |  |  |
| 35 | Priyadharshini KM, George N, Britto DR, Nirmal SR, Tamilarasan M, Kulothungan K. 2021. Assessment of Stress, Resilience, and Coping Style among Medical Students and Effectiveness of Intervention Programs on Stress Level in South India: A Non-randomized Control Trial. Indian Journal of Community Medicine. 46(4):735-738. |  | X | pure psychological data, not psychophysiological data |  |
| 36 | Radamés B, Jorge Vinícius Cestari F, Carina B-M, Pedrolo E, Stela Adami V, Maria de Fátima M. 2018. Stress of nursing students in clinical simulation: a randomized clinical trial. Revista Brasileira de Enfermagem. 71(3):967-974. |  | X | pure psychological data, not psychophysiological data |  |
| 37 | Radcliffe C, Lester H. 2003. Perceived stress during undergraduate medical training: a qualitative study. Medical Education. 37(1):32-38. |  | X | pure psychological data, not psychophysiological data |  |
| 38 | Rafique N, Al-Asoom LI, Latif R, Al Sunni A, Wasi S. 2019. Comparing levels of psychological stress and its inducing factors among medical students. Journal of Taibah University Medical Sciences. 14(6):488-494. |  | X | pure psychological data, not psychophysiological data |  |
| 39 | Rieber N, Betz L, Enck P, Muth E, Nikendei C, Schrauth M, Werner A, Kowalski A, Zipfel S. 2009. Effects of medical training scenarios on heart rate variability and motivation in students and simulated patients. Medical education. 43(6):553-556. | X |  |  |  |
| 40 | Roberts M, Gale T, McGrath J, Wilson M, Roberts MJ, Gale TCE, McGrath JS, Wilson MR. 2016. Rising to the challenge: acute stress appraisals and selection centre performance in applicants to postgraduate specialty training in anaesthesia. Advances in Health Sciences Education. 21(2):323-339. |  | X | pure psychological data, not psychophysiological data |  |
| 41 | Roshanmehr H, Tavakoli R, Khaledi M, Fathi J, Shafiea SM, Aberomand M, Afkhami H, Branson B, Daghagheleh R, Rastegar S. 2021. Evaluating the activity of salivary enzymes as stress biomarkers under psychological stress and their relationship with rumination and personality traits. Biomarkers : biochemical indicators of exposure, response, and susceptibility to chemicals. 26(5):477-482. | X |  |  |  |
| 42 | Rudland JR, Golding C, Jaye C, Tweed M, Wilkinson TJ. 2019. Student belief about the value of challenge. The clinical teacher. 16(4):390-394. |  | X | pure psychological data, not psychophysiological data |  |
| 43 | Rusling M, Masin D, Voss M, Gottumukkala P, Keenan C, Botten M, Chambers D, Parrill C, Dube J, Tucker JR. 2021. Medical student coping and performance in simulated disasters. Anxiety, stress, and coping. 34(6):766-777. |  | X | pure psychological data, not psychophysiological data |  |
| 44 | Schreckengaust R, Littlejohn L, Zarow GJ. 2014. Effects of training and simulated combat stress on leg tourniquet application accuracy, time, and effectiveness. Military Medicine. 179(2):114-120. |  | X | pure psychological data, not psychophysiological data |  |
| 45 | Senturk S, Dogan N. 2018. Determination of the Stress Experienced by Nursing Students' During Nursing Education. International Journal of Caring Sciences. 11(2):896-904. |  | X | pure psychological data, not psychophysiological data |  |
| 46 | Stecz P, Makara-Studzińska M, Białka S, Misiołek H. 2021. Stress responses in high-fidelity simulation among anesthesiology students. Scientific reports. 11(1):17073. | X |  |  |  |
| 47 | Suzuki S, Kumano H, Sakano Y. 2003. Effects of effort and distress coping processes on psychophysiological and psychological stress responses. Int J Psychophysiol. 47(2):117-128. eng. |  | X | Non healthcare / medical education related |  |
| 48 | Tramèr L, Becker C, Hochstrasser S, Marsch S, Hunziker S. 2018. Association of electrocardiogram alterations of rescuers and performance during a simulated cardiac arrest: A prospective simulation study. PloS one. 13(6):e0198661. | X |  |  |  |
| 49 | Tseng T, Iosif AM, Seritan AL. 2011. Stress effects: A study of salivary cortisol levels in third‐year medical students. Stress and Health: Journal of the International Society for the Investigation of Stress. 27(5):436-440. | X |  |  |  |
| 50 | Weaver A. 2011. High-Fidelity Patient Simulation in Nursing Education: An Integrative Review. Nursing Education Perspectives (National League for Nursing). 32(1):37-40. |  | X | pure psychological data, not psychophysiological data |  |
| 51 | Yang H, Thompson C, Bland M. 2012. The effect of clinical experience, judgment task difficulty and time pressure on nurses' confidence calibration in a high-fidelity clinical simulation. BMC Medical Informatics & Decision Making. 12(1):113-113. |  | X | pure psychological data, not psychophysiological data |  |
| 52 | 장현숙, 이정순. 2016. 간호학생의 밀착형 실무진입 임상실습교육이 임상수행능력, 상태불안 및 임상실습 스트레스에 미치는 효과...Effects of Intensive Clinical Training for Nursing Students in Nursing Practice on their Clinical Competence, State Anxiety, and Clinical Practice Stress. Journal of Korean Academy of Fundamentals of Nursing. 23(4):419-429. |  | X | pure psychological data, not psychophysiological data |  |

**52 Articles for further review (DL):**

|  | Article | Include | Exclude | Exclude reason | Unsure |
| --- | --- | --- | --- | --- | --- |
| 1 | Acharya J, Sahani S. 2022. Coping up with Stress as a Medical Student. JNMA; journal of the Nepal Medical Association. 60(248):416-418. |  | X | Not a primary study.  No physiological or psychophysiological data recorded. |  |
| 2 | Al-Ghareeb AZ, Cooper SJ, McKenna LG. 2017. Anxiety and Clinical Performance in Simulated Setting in Undergraduate Health Professionals Education: An Integrative Review. Clinical Simulation In Nursing. 13(10):478-491. |  | X | Integrated review |  |
| 3 | Baker BG, Bhalla A, Doleman B, Yarnold E, Simons S, Lund JN, Williams JP. 2017. Simulation fails to replicate stress in trainees performing a technical procedure in the clinical environment. Medical Teacher. 39(1):53-57. | X |  |  |  |
| 4 | Barbadoro P, Brunzini A, Dolcini J, Formenti L, Luciani A, Messi D, Papetti A, Ponzio E, Germani M, Adrario E. 2023. Stress responses in high-fidelity simulation and standard simulation training among medical students. BMC medical education. 23(1):116. | X |  |  |  |
| 5 | Beltrán-Velasco AI, Bellido-Esteban A, Ruisoto-Palomera P, Clemente-Suárez VJ. 2018. Use of Portable Digital Devices to Analyze Autonomic Stress Response in Psychology Objective Structured Clinical Examination. Journal of Medical Systems. 42(2):35. |  |  |  | X  Study examined students on placement rather than simulation? |
| 6 | Beltrán-Velasco AI, Ruisoto-Palomera P, Bellido-Esteban A, García-Mateos M, Clemente-Suárez VJ. 2019. Analysis of Psychophysiological Stress Response in Higher Education Students Undergoing Clinical Practice Evaluation. Journal of medical systems. 43(3):68. | X |  |  |  |
| 7 | Bialka S, Copik M, Ubych A, Marciniak R, Smereka J, Szarpak L, Misiolek H. 2021. Effect of high-fidelity simulation on alpha-amylase activity and concentrations of secretory immunoglobulin class A, cortisol, and testosterone among medical students. Endocrine. 73(2):431-438. | X |  |  |  |
| 8 | Brasil GdC, Lima LTB, Cunha EC, Cruz FOdAMd, Ribeiro LM. 2021. Stress level experienced by participants in realistic simulation: a systematic review. Revista Brasileira de Enfermagem. 74. |  | X | Systematic review |  |
| 9 | Buller MJ, Welles AP, Friedl KE. 2018. Wearable physiological monitoring for human thermal-work strain optimization. J Appl Physiol (1985). 124(2):432-441. eng. |  | X | Not related to higher education or equivalent students |  |
| 10 | Demaria S, Jr., Bryson EO, Mooney TJ, Silverstein JH, Reich DL, Bodian C, Levine AI. 2010. Adding emotional stressors to training in simulated cardiopulmonary arrest enhances participant performance. Medical education. 44(10):1006-1015. | X |  |  |  |
| 11 | DeMaria S, Silverman ER, Lapidus KAB, Williams CH, Spivack J, Levine A, Goldberg A. 2016. The impact of simulated patient death on medical students' stress response and learning of ACLS. Medical teacher. 38(7):730-737. | X |  |  |  |
| 12 | Feeley AA, Feeley IH, McManus R, Lunn JV, Sheehan E, Merghani K. 2022. Evaluating the Impact of Supervision on Surgical Trainees Stress Response During Simulated Surgical Procedures; A Crossover Randomized Trial. Journal of Surgical Education. 79(6):1379-1386. | X |  |  |  |
| 13 | Flinn JT, Miller A, Pyatka N, Brewer J, Schneider T, Cao CGL. 2016. The effect of stress on learning in surgical skill acquisition. Medical teacher. 38(9):897-903. | X |  |  |  |
| 14 | Georgiou K, Larentzakis A, Papavassiliou AG. 2017. Surgeons' and surgical trainees' acute stress in real operations or simulation: A systematic review. The Surgeon. 15(6):355-365. |  | X | Systematic review |  |
| 15 | Gürdil Yilmaz S, Yıldız Karadeniz E, dem Lafçi D. 2022. Clinical‐practice stress levels and factors affecting these on first‐year nursing students. Perspectives in Psychiatric Care. 58(4):3009-3015. |  | X | No physiological or psychophysiological data recorded. |  |
| 16 | Harvey A, Nathens AB, Bandiera G, LeBlanc VR. 2010. Threat and challenge: cognitive appraisal and stress responses in simulated trauma resuscitations. Medical Education. 44(6):587-594. |  | X | Participants were not students |  |
| 17 | Hogg G, Miller D. 2016. The effects of an enhanced simulation programme on medical students' confidence responding to clinical deterioration. BMC medical education. 16:161. |  | X | No physiological or psychophysiological data recorded. |  |
| 18 | Judd BK, Alison JA, Waters D, Gordon CJ. 2016. Comparison of Psychophysiological Stress in Physiotherapy Students Undertaking Simulation and Hospital-Based Clinical Education. Simulation in Healthcare. 11(4):271-277. | X |  |  |  |
| 19 | Keitel A, Ringleb M, Schwartges I, Weik U, Picker O, Stockhorst U, Deinzer R. 2011. Endocrine and psychological stress responses in a simulated emergency situation. Psychoneuroendocrinology. 36(1):98-108. | X |  |  |  |
| 20 | Kharasch M, Aitchison P, Pettineo C, Pettineo L, Wang EE. 2011. Physiological Stress Responses of Emergency Medicine Residents During an Immersive Medical Simulation Scenario. Disease-a-Month. 57(11):700-705. |  | X | Participants were not students |  |
| 21 | Łoś K, Chmielewski J, Cebula G, Bielecki T, Torres K, Łuczyński W. 2021. Relationship between mindfulness, stress, and performance in medical students in pediatric emergency simulations. GMS journal for medical education. 38(4):Doc78. | X |  |  |  |
| 22 | Macdougall L, Martin R, McCallum I, Grogan E. 2013. Simulation and stress: acceptable to students and not confidence-busting. The clinical teacher. 10(1):38-41. |  | X | No physiological or psychophysiological data recorded.  Questionable ethics (authors stated not required which I disagree with) |  |
| 23 | MacQuarrie AS, Hunter JR, Sheridan S, Hlushak A, Sutton C, Wickham J. 2022. Paramedic Student Clinical Performance During High-Fidelity Simulation After a Physically Demanding Occupational Task: A Pilot Randomized Crossover Trial. Simulation in Healthcare. 17(4):234-241. eng. | X |  |  |  |
| 24 | Martín-Rodríguez F, Castro Villamor MA, López-Izquierdo R, Portillo Rubiales RM, Ortega GJ, Sanz-García A. 2021. Can anxiety in undergraduate students in a high-fidelity clinical simulation be predicted? A randomized, sham-controlled, blinded trial. Nurse Educ Today. 98:104774. eng. | X |  |  |  |
| 25 | McKay KAC, Buen JE, Bohan KJ, Maye JP. 2010. Determining the Relationship of Acute Stress, Anxiety, and Salivary α-Amylase Level With Performance of Student Nurse Anesthetists During Human-Based Anesthesia Simulator Training. AANA Journal. 78(4):301-309. | X |  |  |  |
| 26 | McKerrow I, Carney PA, Caretta-Weyer H, Furnari M, Miller Juve A. 2020. Trends in medical students' stress, physical, and emotional health throughout training. Medical education online. 25(1):1709278. |  | X | No physiological or psychophysiological data recorded. |  |
| 27 | Mendes SS, Salvi CPP, Moraes BFM, De Martino MMF. 2019. INSTRUMENTS FOR THE EVALUATION OF STRESS IN NURSING STUDENTS. Journal of Nursing UFPE / Revista de Enfermagem UFPE. 13(3):829-838. |  | X | Bibliographic study |  |
| 28 | Morrell N, Ridgway V. 2014. Are we preparing student nurses for final practice placement? British Journal of Nursing. 23(10):518-523. |  | X | Qualitative study |  |
| 29 | Mueller G, Moloff A, Wedmore I, Schoeff J, LaPorta AJ. 2012. High intensity scenario training of military medical students to increase learning capacity and management of stress response. Journal of special operations medicine : a peer reviewed journal for SOF medical professionals. 12(2):71-76. |  | X | No physiological or psychophysiological data recorded. |  |
| 30 | Nachiappan M, Bikramjit P, Aung WT, Htoo HKS, Sudipta P. 2020. The impact of stressors on the learning outcome of high-fidelity patient simulation in undergraduate medical students. The Medical journal of Malaysia. 75(3):209-215. |  | X | No physiological or psychophysiological data recorded. |  |
| 31 | Nakayama N, Arakawa N, Ejiri H, Matsuda R, Makino T. 2018. Heart rate variability can clarify students' level of stress during nursing simulation. PLoS ONE. 13(4). | X |  |  |  |
| 32 | Norouzi N, Imani B. 2021. Clinical Education Stressors in Operating Room Students: A Qualitative Study. Investigacion & Educacion en Enfermeria. 39(1):97-108. |  | X | Qualitative study |  |
| 33 | Palekar TJ, Mokashi MG, Anwer S, Kakrani AL, Khandare SD, Alghadir AH. 2015. Effect of Galvanic Skin Resistance-Aided Biofeedback Training in Reducing the Pulse Rate, Respiratory Rate, and Blood Pressure Due to Perceived Stress in Physiotherapy Students. / Fizyoterapi Öğrencilerinde Algılanan Strese Bağlı Nabız Hızı, Solunum Hızı ve Kan Basıncının Azaltılmasında Galvanik Deri Direnci Destekli Bio-Geri Bildirim Eğitiminin Etkisi. Turkish Journal of Physical Medicine & Rehabilitation / Turkiye Fiziksel Tip ve Rehabilitasyon Dergisi. 61(2):116-119. | X |  |  |  |
| 34 | Park HJ, Choi D, Park HA, Lee CA. 2022. Nurse evaluation of stress levels during CPR training with heart rate variability using smartwatches according to their personality: A prospective, observational study. PLoS ONE. 17(6). | X |  |  |  |
| 35 | Priyadharshini KM, George N, Britto DR, Nirmal SR, Tamilarasan M, Kulothungan K. 2021. Assessment of Stress, Resilience, and Coping Style among Medical Students and Effectiveness of Intervention Programs on Stress Level in South India: A Non-randomized Control Trial. Indian Journal of Community Medicine. 46(4):735-738. |  | X | No physiological or psychophysiological data recorded. |  |
| 36 | Radamés B, Jorge Vinícius Cestari F, Carina B-M, Pedrolo E, Stela Adami V, Maria de Fátima M. 2018. Stress of nursing students in clinical simulation: a randomized clinical trial. Revista Brasileira de Enfermagem. 71(3):967-974. |  | X | No physiological or psychophysiological data recorded. |  |
| 37 | Radcliffe C, Lester H. 2003. Perceived stress during undergraduate medical training: a qualitative study. Medical Education. 37(1):32-38. |  | X | Qualitative study |  |
| 38 | Rafique N, Al-Asoom LI, Latif R, Al Sunni A, Wasi S. 2019. Comparing levels of psychological stress and its inducing factors among medical students. Journal of Taibah University Medical Sciences. 14(6):488-494. |  | X | No physiological or psychophysiological data recorded. |  |
| 39 | Rieber N, Betz L, Enck P, Muth E, Nikendei C, Schrauth M, Werner A, Kowalski A, Zipfel S. 2009. Effects of medical training scenarios on heart rate variability and motivation in students and simulated patients. Medical education. 43(6):553-556. | X |  |  |  |
| 40 | Roberts M, Gale T, McGrath J, Wilson M, Roberts MJ, Gale TCE, McGrath JS, Wilson MR. 2016. Rising to the challenge: acute stress appraisals and selection centre performance in applicants to postgraduate specialty training in anaesthesia. Advances in Health Sciences Education. 21(2):323-339. |  | X | No physiological or psychophysiological data recorded. |  |
| 41 | Roshanmehr H, Tavakoli R, Khaledi M, Fathi J, Shafiea SM, Aberomand M, Afkhami H, Branson B, Daghagheleh R, Rastegar S. 2021. Evaluating the activity of salivary enzymes as stress biomarkers under psychological stress and their relationship with rumination and personality traits. Biomarkers : biochemical indicators of exposure, response, and susceptibility to chemicals. 26(5):477-482. |  | X | Does not include a clinical scenario/simulation (Participants sat an exam). |  |
| 42 | Rudland JR, Golding C, Jaye C, Tweed M, Wilkinson TJ. 2019. Student belief about the value of challenge. The clinical teacher. 16(4):390-394. |  | X | No physiological or psychophysiological data recorded. |  |
| 43 | Rusling M, Masin D, Voss M, Gottumukkala P, Keenan C, Botten M, Chambers D, Parrill C, Dube J, Tucker JR. 2021. Medical student coping and performance in simulated disasters. Anxiety, stress, and coping. 34(6):766-777. |  | X | No physiological or psychophysiological data recorded. |  |
| 44 | Schreckengaust R, Littlejohn L, Zarow GJ. 2014. Effects of training and simulated combat stress on leg tourniquet application accuracy, time, and effectiveness. Military Medicine. 179(2):114-120. |  | X | No physiological or psychophysiological data recorded. |  |
| 45 | Senturk S, Dogan N. 2018. Determination of the Stress Experienced by Nursing Students' During Nursing Education. International Journal of Caring Sciences. 11(2):896-904. |  | X | No physiological or psychophysiological data recorded. |  |
| 46 | Stecz P, Makara-Studzińska M, Białka S, Misiołek H. 2021. Stress responses in high-fidelity simulation among anesthesiology students. Scientific reports. 11(1):17073. | X |  |  |  |
| 47 | Suzuki S, Kumano H, Sakano Y. 2003. Effects of effort and distress coping processes on psychophysiological and psychological stress responses. Int J Psychophysiol. 47(2):117-128. eng. |  | X | Participants had to complete arithmetic |  |
| 48 | Tramèr L, Becker C, Hochstrasser S, Marsch S, Hunziker S. 2018. Association of electrocardiogram alterations of rescuers and performance during a simulated cardiac arrest: A prospective simulation study. PloS one. 13(6):e0198661. | X |  |  |  |
| 49 | Tseng T, Iosif AM, Seritan AL. 2011. Stress effects: A study of salivary cortisol levels in third‐year medical students. Stress and Health: Journal of the International Society for the Investigation of Stress. 27(5):436-440. |  |  |  | X  Study examined students on placement rather than simulation? |
| 50 | Weaver A. 2011. High-Fidelity Patient Simulation in Nursing Education: An Integrative Review. Nursing Education Perspectives (National League for Nursing). 32(1):37-40. |  | X | Integrated review |  |
| 51 | Yang H, Thompson C, Bland M. 2012. The effect of clinical experience, judgment task difficulty and time pressure on nurses' confidence calibration in a high-fidelity clinical simulation. BMC Medical Informatics & Decision Making. 12(1):113-113. | X |  |  |  |
| 52 | 장현숙, 이정순. 2016. 간호학생의 밀착형 실무진입 임상실습교육이 임상수행능력, 상태불안 및 임상실습 스트레스에 미치는 효과...Effects of Intensive Clinical Training for Nursing Students in Nursing Practice on their Clinical Competence, State Anxiety, and Clinical Practice Stress. Journal of Korean Academy of Fundamentals of Nursing. 23(4):419-429. |  | X | No physiological or psychophysiological data recorded. |  |

**Seven articles where consensus not achieved – for further review (PH):**

|  | Article | Include | Exclude | Exclude reason | Unsure |
| --- | --- | --- | --- | --- | --- |
| 5 | Beltrán-Velasco AI, Bellido-Esteban A, Ruisoto-Palomera P, Clemente-Suárez VJ. 2018. Use of Portable Digital Devices to Analyze Autonomic Stress Response in Psychology Objective Structured Clinical Examination. Journal of Medical Systems. 42(2):35. | X |  |  |  |
| 12 | Feeley AA, Feeley IH, McManus R, Lunn JV, Sheehan E, Merghani K. 2022. Evaluating the Impact of Supervision on Surgical Trainees Stress Response During Simulated Surgical Procedures; A Crossover Randomized Trial. Journal of Surgical Education. 79(6):1379-1386. | X |  |  |  |
| 16 | Harvey A, Nathens AB, Bandiera G, LeBlanc VR. 2010. Threat and challenge: cognitive appraisal and stress responses in simulated trauma resuscitations. Medical Education. 44(6):587-594. | X |  |  |  |
| 18 | Judd BK, Alison JA, Waters D, Gordon CJ. 2016. Comparison of Psychophysiological Stress in Physiotherapy Students Undertaking Simulation and Hospital-Based Clinical Education. Simulation in Healthcare. 11(4):271-277. | X |  |  |  |
| 41 | Roshanmehr H, Tavakoli R, Khaledi M, Fathi J, Shafiea SM, Aberomand M, Afkhami H, Branson B, Daghagheleh R, Rastegar S. 2021. Evaluating the activity of salivary enzymes as stress biomarkers under psychological stress and their relationship with rumination and personality traits. Biomarkers : biochemical indicators of exposure, response, and susceptibility to chemicals. 26(5):477-482. |  | X | This study evaluated the stress involved in undertaking “principally written exams”, rather than clinical scenarios/simulations |  |
| 49 | Tseng T, Iosif AM, Seritan AL. 2011. Stress effects: A study of salivary cortisol levels in third‐year medical students. Stress and Health: Journal of the International Society for the Investigation of Stress. 27(5):436-440. |  | X | The study looked at stress markers during actual medical practice verses while students were on holiday, rather than in a simulated clinical scenario |  |
| 51 | Yang H, Thompson C, Bland M. 2012. The effect of clinical experience, judgment task difficulty and time pressure on nurses' confidence calibration in a high-fidelity clinical simulation. BMC Medical Informatics & Decision Making. 12(1):113-113. |  | X | The study explored individual confidence/cognitive bias via a self-score without any physiological measurement. |  |

**Appendix 3: Articles excluded with reasons**

Articles **EXCLUDED** (numbers relate to list of 52, not reference list numbering)**:**

Studies that gathered pure psychological data, not psychophysiological data (17)

- 1 Acharya J, Sahani S. 2022. Coping up with Stress as a Medical Student. JNMA; journal of the Nepal Medical Association. 60(248):416-418.
- 2 Al-Ghareeb AZ, Cooper SJ, McKenna LG. 2017. Anxiety and Clinical Performance in Simulated Setting in Undergraduate Health Professionals Education: An Integrative Review. Clinical Simulation In Nursing. 13(10):478-491.
- 15 Gürdil Yilmaz S, Yıldız Karadeniz E, dem Lafçi D. 2022. Clinical‐practice stress levels and factors affecting these on first‐year nursing students. Perspectives in Psychiatric Care. 58(4):3009-3015.
- 17 Hogg G, Miller D. 2016. The effects of an enhanced simulation programme on medical students' confidence responding to clinical deterioration. BMC medical education. 16:161.
- 22 Macdougall L, Martin R, McCallum I, Grogan E. 2013. Simulation and stress: acceptable to students and not confidence-busting. The clinical teacher. 10(1):38-41.
- 26 McKerrow I, Carney PA, Caretta-Weyer H, Furnari M, Miller Juve A. 2020. Trends in medical students' stress, physical, and emotional health throughout training. Medical education online. 25(1):1709278.
- 29 Mueller G, Moloff A, Wedmore I, Schoeff J, LaPorta AJ. 2012. High intensity scenario training of military medical students to increase learning capacity and management of stress response. Journal of special operations medicine : a peer reviewed journal for SOF medical professionals. 12(2):71-76.
- 30 Nachiappan M, Bikramjit P, Aung WT, Htoo HKS, Sudipta P. 2020. The impact of stressors on the learning outcome of high-fidelity patient simulation in undergraduate medical students. The Medical journal of Malaysia. 75(3):209-215.
- 35 Priyadharshini KM, George N, Britto DR, Nirmal SR, Tamilarasan M, Kulothungan K. 2021. Assessment of Stress, Resilience, and Coping Style among Medical Students and Effectiveness of Intervention Programs on Stress Level in South India: A Non-randomized Control Trial. Indian Journal of Community Medicine. 46(4):735-738.
- 36 Radamés B, Jorge Vinícius Cestari F, Carina B-M, Pedrolo E, Stela Adami V, Maria de Fátima M. 2018. Stress of nursing students in clinical simulation: a randomized clinical trial. Revista Brasileira de Enfermagem. 71(3):967-974.
- 38 Rafique N, Al-Asoom LI, Latif R, Al Sunni A, Wasi S. 2019. Comparing levels of psychological stress and its inducing factors among medical students. Journal of Taibah University Medical Sciences. 14(6):488-494.
- 40 Roberts M, Gale T, McGrath J, Wilson M, Roberts MJ, Gale TCE, McGrath JS, Wilson MR. 2016. Rising to the challenge: acute stress appraisals and selection centre performance in applicants to postgraduate specialty training in anaesthesia. Advances in Health Sciences Education. 21(2):323-339.
- 42 Rudland JR, Golding C, Jaye C, Tweed M, Wilkinson TJ. 2019. Student belief about the value of challenge. The clinical teacher. 16(4):390-394.
- 43 Rusling M, Masin D, Voss M, Gottumukkala P, Keenan C, Botten M, Chambers D, Parrill C, Dube J, Tucker JR. 2021. Medical student coping and performance in simulated disasters. Anxiety, stress, and coping. 34(6):766-777.
- 44 Schreckengaust R, Littlejohn L, Zarow GJ. 2014. Effects of training and simulated combat stress on leg tourniquet application accuracy, time, and effectiveness. Military Medicine. 179(2):114-120.
- 45 Senturk S, Dogan N. 2018. Determination of the Stress Experienced by Nursing Students' During Nursing Education. International Journal of Caring Sciences. 11(2):896-904.
- 52 장현숙, 이정순. 2016. 간호학생의 밀착형 실무진입 임상실습교육이 임상수행능력, 상태불안 및 임상실습 스트레스에 미치는 효과...Effects of Intensive Clinical Training for Nursing Students in Nursing Practice on their Clinical Competence, State Anxiety, and Clinical Practice Stress. Journal of Korean Academy of Fundamentals of Nursing. 23(4):419-429.

Pure qualitative studies with no quantitative (objectively recorded) data (5)

- 27 Mendes SS, Salvi CPP, Moraes BFM, De Martino MMF. 2019. INSTRUMENTS FOR THE EVALUATION OF STRESS IN NURSING STUDENTS. Journal of Nursing UFPE / Revista de Enfermagem UFPE. 13(3):829-838.
- 28 Morrell N, Ridgway V. 2014. Are we preparing student nurses for final practice placement? British Journal of Nursing. 23(10):518-523.
- 32 Norouzi N, Imani B. 2021. Clinical Education Stressors in Operating Room Students: A Qualitative Study. Investigacion & Educacion en Enfermeria. 39(1):97-108.
- 37 Radcliffe C, Lester H. 2003. Perceived stress during undergraduate medical training: a qualitative study. Medical Education. 37(1):32-38.
- 51 Yang H, Thompson C, Bland M. 2012. The effect of clinical experience, judgment task difficulty and time pressure on nurses' confidence calibration in a high-fidelity clinical simulation. BMC Medical Informatics & Decision Making. 12(1):113-113.

Non healthcare / medical education related (can include military medic training if relatable) (5)

- 9 Buller MJ, Welles AP, Friedl KE. 2018. Wearable physiological monitoring for human thermal-work strain optimization. J Appl Physiol (1985). 124(2):432-441. eng.
- 20 Kharasch M, Aitchison P, Pettineo C, Pettineo L, Wang EE. 2011. Physiological Stress Responses of Emergency Medicine Residents During an Immersive Medical Simulation Scenario. Disease-a-Month. 57(11):700-705.
- 41 Roshanmehr H, Tavakoli R, Khaledi M, Fathi J, Shafiea SM, Aberomand M, Afkhami H, Branson B, Daghagheleh R, Rastegar S. 2021. Evaluating the activity of salivary enzymes as stress biomarkers under psychological stress and their relationship with rumination and personality traits. Biomarkers : biochemical indicators of exposure, response, and susceptibility to chemicals. 26(5):477-482.
- 47 Suzuki S, Kumano H, Sakano Y. 2003. Effects of effort and distress coping processes on psychophysiological and psychological stress responses. Int J Psychophysiol. 47(2):117-128. eng.
- 49 Tseng T, Iosif AM, Seritan AL. 2011. Stress effects: A study of salivary cortisol levels in third‐year medical students. Stress and Health: Journal of the International Society for the Investigation of Stress. 27(5):436-440.

Other literature (systematic or integrated) reviews – these should be further examined for primary sources (exclude articles prior to 2000) (3)

- 8 Brasil GdC, Lima LTB, Cunha EC, Cruz FOdAMd, Ribeiro LM. 2021. Stress level experienced by participants in realistic simulation: a systematic review. Revista Brasileira de Enfermagem. 74.
- 14 Georgiou K, Larentzakis A, Papavassiliou AG. 2017. Surgeons' and surgical trainees' acute stress in real operations or simulation: A systematic review. The Surgeon. 15(6):355-365.
- 50 Weaver A. 2011. High-Fidelity Patient Simulation in Nursing Education: An Integrative Review. Nursing Education Perspectives (National League for Nursing). 32(1):37-40.

**Appendix 4: MMAT Quality assessment results**

| **Author and year** | **Title** | **Study design** | **Are there clear research questions?** | **Do the collected data allow to address the research questions?** | **Qualitative**  **1.1**  **1.2**  **1.3**  **1.4**  **1.5** | **Quantitative randomized controlled trials**  **2.1**  **2.2**  **2.3**  **2.4**  **2.5** | **Quantitative non- randomized**  **3.1**  **3.2**  **3.3**  **3.4**  **3.5** | **Quantitative descriptive**  **4.1**  **4.2**  **4.3**  **4.4**  **4.5** | **Mixed methods**  **5.1**  **5.2**  **5.3**  **5.4**  **5.5** | **Overall Score.**  **‘Yes’ was given a nominal value of 2, ‘No’ a value of 0, and ‘Unclear/can't tell’ a value of 1** |
| --- | --- | --- | --- | --- | --- | --- | --- | --- | --- | --- |
| **[37]** Baker BG, Bhalla A, Doleman B, Yarnold E, Simons S, Lund JN, Williams JP. 2017 | Simulation fails to replicate stress in trainees performing a technical procedure in the clinical environment | Mixed methods | Yes | Yes | Yes | No | No | Yes | Yes | 10 |
| **[26]** Barbadoro P, Brunzini A, Dolcini J, Formenti L, Luciani A, Messi D, Papetti A, Ponzio E, Germani M, Adrario E. 2023. | Stress responses in high-fidelity simulation and standard simulation training among medical students | Mixed methods | Yes | Yes | Yes | No | Yes | Yes | Yes | 12 |
| **[45]** Beltrán-Velasco AI, Bellido-Esteban A, Ruisoto-Palomera P, Clemente-Suárez VJ. 2018. | Use of Portable Digital Devices to Analyze Autonomic Stress Response in Psychology Objective Structured Clinical Examination | Quantitative research | Yes | Yes | No | No | Can’t tell | Yes | No | 7 |
| **[42]** Beltrán-Velasco AI, Ruisoto-Palomera P, Bellido-Esteban A, García-Mateos M, Clemente-Suárez VJ. 2019. | Analysis of Psychophysiological Stress Response in Higher Education Students Undergoing Clinical Practice Evaluation | Mixed methods | Yes | Yes | Yes | No | Yes | Yes | Yes | 12 |
| **[27]** Bialka S, Copik M, Ubych A, Marciniak R, Smereka J, Szarpak L, Misiolek H. 2021. | Effect of high-fidelity simulation on alpha-amylase activity and concentrations of secretory immunoglobulin class A, cortisol, and testosterone among medical students | Quantitative research | Yes | Yes | No | No | Can’t tell | Yes | No | 7 |
| **[28]** Demaria S, Jr., Bryson EO, Mooney TJ, Silverstein JH, Reich DL, Bodian C, Levine AI. 2010. | Adding emotional stressors to training in simulated cardiopulmonary arrest enhances participant performance | Mixed methods | Yes | Yes | Yes | Yes | No | Yes | Yes | 12 |
| **[29]** DeMaria S, Silverman ER, Lapidus KAB, Williams CH, Spivack J, Levine A, Goldberg A. 2016 | The impact of simulated patient death on medical students’ stress response and learning of ACLS | Quantitative research | Yes | Yes | No | Yes | No | Yes | No | 8 |
| **[30]** Feeley AA, Feeley IH, McManus R, Lunn JV, Sheehan E, Merghani K. 2022. | Evaluating the Impact of Supervision on Surgical Trainees Stress Response During Simulated Surgical Procedures; A Crossover Randomized Trial | Quantitative research | Yes | Yes | No | Yes | No | Yes | No | 8 |
| **[31]** Flinn JT, Miller A, Pyatka N, Brewer J, Schneider T, Cao CGL. 2016. | The effect of stress on learning in surgical skill acquisition | Mixed methods | Yes | Yes | Yes | Yes | No | Yes | Yes | 12 |
| **[7]** Harvey A, Nathens AB, Bandiera G, LeBlanc VR. 2010. | Threat and challenge: cognitive appraisal and stress responses in simulated trauma resuscitations | Mixed methods | Yes | Yes | Yes | Yes | No | Yes | Yes | 12 |
| **[43]** Judd BK, Alison JA, Waters D, Gordon CJ. 2016. | Comparison of Psychophysiological Stress in Physiotherapy Students Undertaking Simulation and Hospital-Based Clinical Education | Mixed methods | Yes | Yes | Yes | No | Can’t ell | Yes | Yes | 11 |
| **[32]** Keitel A, Ringleb M, Schwartges I, Weik U, Picker O, Stockhorst U, Deinzer R. 2011. | Endocrine and psychological stress responses in a simulated emergency situation | Mixed methods | Yes | Yes | Yes | No | No | Yes | Yes | 10 |
| **[33]** Los, K, Chmielewski J, Cebula G, Bielecki T, Torres K, Luczynski, W. 2021. | Relationship between mindfulness, stress, and performance in medical students in pediatric emergency simulations | Mixed methods | Yes | Yes | Yes | No | Can’t tell | Yes | Yes | 11 |
| **[25]** MacQuarrie AS, Hunter JR, Sheridan S, Hlushak A, Sutton C, Wickham J. 2022. | Paramedic Student Clinical Performance During High-Fidelity Simulation After a Physically Demanding Occupational Task | Quantitative research | Yes | Yes | No | Yes | No | Yes | No | 8 |
| **[34]** Martín-Rodríguez F, Castro Villamor MA, López-Izquierdo R, Portillo Rubiales RM, Ortega GJ, Sanz-García A. 2021 | Can anxiety in undergraduate students in a high-fidelity clinical simulation be predicted? A randomized, sham-controlled, blinded trial | Mixed methods | Yes | Yes | Yes | Yes | No | Yes | Yes | 12 |
| **[38]** McKay KAC, Buen JE, Bohan KJ, Maye JP. 2010. | Determining the Relationship of Acute Stress, Anxiety, and Salivary α-Amylase Level With Performance of Student Nurse Anesthetists During Human-Based Anesthesia Simulator Training | Mixed methods | Yes | Yes | Yes | No | No | Yes | Yes | 10 |
| **[39]** Nakayama N, Arakawa N, Ejiri H, Matsuda R, Makino T. 2018. | Heart rate variability can clarify students’ level of stress during nursing simulation | Mixed methods | Yes | Yes | Yes | No | No | Yes | Yes | 10 |
| **[44]** Palekar TJ, Mokashi MG, Anwer S, Kakrani AL, Khandare SD, Alghadir AH. 2015 | Effect of Galvanic Skin Resistance-Aided Biofeedback Training in Reducing the Pulse Rate, Respiratory Rate, and Blood Pressure Due to Perceived Stress in Physiotherapy Students | Mixed methods | Yes | Yes | Yes | No | No | Yes | Yes | 10 |
| **[40]** Park HJ, Choi D, Park HA, Lee CA. 2022. | Nurse evaluation of stress levels during CPR training with heart rate variability using smartwatches according to their personality: A prospective, observational study | Mixed methods | Yes | Yes | Yes | No | Can’t tell | Yes | Yes | 10 |
| **[35]** Rieber N, Betz L, Enck P, Muth E, Nikendei C, Schrauth M, Werner A, Kowalski A, Zipfel S. 2009. | Effects of medical training scenarios on heart rate variability and motivation in students and simulated patients | Mixed methods | Yes | Yes | Yes | No | Yes | Yes | Yes | 12 |
| **[41]** Stecz P, Makara-Studzińska M, Białka S, Misiołek H. 2021. | Stress responses in high‐fidelity simulation among anesthesiology students | Mixed methods | Yes | Yes | Yes | No | Yes | Yes | Yes | 12 |
| **[36]** Tramèr L, Becker C, Hochstrasser S, Marsch S, Hunziker S. 2018. | Association of electrocardiogram alterations of rescuers and performance during a simulated cardiac arrest: A prospective simulation study | Quantitative research | Yes | Yes | No | No | Can’t tell | Yes | No | 7 |

**Appendix 5: Preferred Reporting Items for Systematic reviews and Meta-Analyses extension for Scoping Reviews (PRISMA-ScR) Checklist**

| **SECTION** | **ITEM** | **PRISMA-ScR CHECKLIST ITEM** | **REPORTED ON PAGE #** |
| --- | --- | --- | --- |
| **TITLE** | | | |
| Title | 1 | Identify the report as a scoping review. | 1 |
| **ABSTRACT** | | | |
| Structured summary | 2 | Provide a structured summary that includes (as applicable): background, objectives, eligibility criteria, sources of evidence, charting methods, results, and conclusions that relate to the review questions and objectives. | 1 |
| **INTRODUCTION** | | | |
| Rationale | 3 | Describe the rationale for the review in the context of what is already known. Explain why the review questions/objectives lend themselves to a scoping review approach. | 2-5 |
| Objectives | 4 | Provide an explicit statement of the questions and objectives being addressed with reference to their key elements (e.g., population or participants, concepts, and context) or other relevant key elements used to conceptualize the review questions and/or objectives. | 5-6 |
| **METHODS** | | | |
| Protocol and registration | 5 | Indicate whether a review protocol exists; state if and where it can be accessed (e.g., a Web address); and if available, provide registration information, including the registration number. | 5 and Appendix 1 |
| Eligibility criteria | 6 | Specify characteristics of the sources of evidence used as eligibility criteria (e.g., years considered, language, and publication status), and provide a rationale. | 6-7 and Appendix 1 |
| Information sources* | 7 | Describe all information sources in the search (e.g., databases with dates of coverage and contact with authors to identify additional sources), as well as the date the most recent search was executed. | 6-8 and Appendix 1 |
| Search | 8 | Present the full electronic search strategy for at least 1 database, including any limits used, such that it could be repeated. | Appendix 1 |
| Selection of sources of evidence† | 9 | State the process for selecting sources of evidence (i.e., screening and eligibility) included in the scoping review. | 8-9 and Appendix 4 |
| Data charting process‡ | 10 | Describe the methods of charting data from the included sources of evidence (e.g., calibrated forms or forms that have been tested by the team before their use, and whether data charting was done independently or in duplicate) and any processes for obtaining and confirming data from investigators. | 7 and Appendix 4 |
| Data items | 11 | List and define all variables for which data were sought and any assumptions and simplifications made. | Appendix 1 |
| Critical appraisal of individual sources of evidence§ | 12 | If done, provide a rationale for conducting a critical appraisal of included sources of evidence; describe the methods used and how this information was used in any data synthesis (if appropriate). | 7-8 and Appendix 4 |
| Synthesis of results | 13 | Describe the methods of handling and summarizing the data that were charted. | 7, Figure 1 and Appendix 4 |
| **RESULTS** | | | |
| Selection of sources of evidence | 14 | Give numbers of sources of evidence screened, assessed for eligibility, and included in the review, with reasons for exclusions at each stage, ideally using a flow diagram. | 8: Figure 1, Appendix 2 and 3 |
| Characteristics of sources of evidence | 15 | For each source of evidence, present characteristics for which data were charted and provide the citations. | Table 1, 14-17 |
| Critical appraisal within sources of evidence | 16 | If done, present data on critical appraisal of included sources of evidence (see item 12). | Appendix 4 |
| Results of individual sources of evidence | 17 | For each included source of evidence, present the relevant data that were charted that relate to the review questions and objectives. | 14-17 |
| Synthesis of results | 18 | Summarize and/or present the charting results as they relate to the review questions and objectives. | 7, 14-17 |
| **DISCUSSION** | | | |
| Summary of evidence | 19 | Summarize the main results (including an overview of concepts, themes, and types of evidence available), link to the review questions and objectives, and consider the relevance to key groups. | 18-20 |
| Limitations | 20 | Discuss the limitations of the scoping review process. | 20-21 |
| Conclusions | 21 | Provide a general interpretation of the results with respect to the review questions and objectives, as well as potential implications and/or next steps. | 22 |
| **FUNDING** | | | |
| Funding | 22 | Describe sources of funding for the included sources of evidence, as well as sources of funding for the scoping review. Describe the role of the funders of the scoping review. | NA |

JBI = Joanna Briggs Institute; PRISMA-ScR = Preferred Reporting Items for Systematic reviews and Meta-Analyses extension for Scoping Reviews.

* Where *sources of evidence* (see second footnote) are compiled from, such as bibliographic databases, social media platforms, and Web sites.

† A more inclusive/heterogeneous term used to account for the different types of evidence or data sources (e.g., quantitative and/or qualitative research, expert opinion, and policy documents) that may be eligible in a scoping review as opposed to only studies. This is not to be confused with *information sources* (see first footnote).

‡ The frameworks by Arksey and O’Malley (6) and Levac and colleagues (7) and the JBI guidance (4, 5) refer to the process of data extraction in a scoping review as data charting*.*

§ The process of systematically examining research evidence to assess its validity, results, and relevance before using it to inform a decision. This term is used for items 12 and 19 instead of "risk of bias" (which is more applicable to systematic reviews of interventions) to include and acknowledge the various sources of evidence that may be used in a scoping review (e.g., quantitative and/or qualitative research, expert opinion, and policy document).

*From:* Tricco AC, Lillie E, Zarin W, O'Brien KK, Colquhoun H, Levac D, et al. PRISMA Extension for Scoping Reviews (PRISMAScR): Checklist and Explanation. Ann Intern Med. 2018;169:467–473. [doi: 10.7326/M18-0850](http://annals.org/aim/fullarticle/2700389/prisma-extension-scoping-reviews-prisma-scr-checklist-explanation).
